# Supplementary material for: Variable absorption of mutational trends by prion-forming domains during Saccharomycetes evolution
Source: PeerJ. 2020 Aug 6;8:e9669. doi: 10.7717/peerj.9669 (PMC7415223; doi:10.7717/peerj.9669)
Supplement: Supplemental Information 2 [file peerj-08-9669-s002.docx]

| **UniProt Accession ID** | **UniProt name** | **# of orthologs** | **(1)*** | **(2)*** |
| --- | --- | --- | --- | --- |
| Q12221  P38216  P09547  P38180  P40956  Q06449  P25367  Q99383  P05453  P40356  P32588  P14922  P53894  P32770  Q02629  Q05672  P54785  P40070  Q08972  P32432  Q12224  P18494  P32831  Q05166  P14907  P23202  Q08925  P38691  P12383  Q12139  P38429  Q12361 | PUF2_YEAST  YBM6_YEAST  SWI1_YEAST  YBI1_YEAST  GTS1_YEAST  PIN3_YEAST  RNQ1_YEAST  HRP1_YEAST  ERF3_YEAST  MED3_YEAST  PUB1_YEAST  CYC8_YEAST  CBK1_YEAST  NRP1_YEAST  NU100_YEAST  RBS1_YEAST  MOT3_YEAST  LSM4_YEAST  NEW1_YEAST  SFP1_YEAST  RLM1_YEAST  GLN3_YEAST  NGR1_YEAST  NUP59_YEAST  NSP1_YEAST  URE2_YEAST  MRN1_YEAST  KSP1_YEAST  PDR1_YEAST  YP022_YEAST  SAP30_YEAST  GPR1_YEAST | 9  4  56  27  58  55  26  57  62  27  62  61  59  63  11  52  25  59  63  64  30  58  55  54  57  66  65  59  22  51  65  59 | 1  1  0.982143  0.962963  0.948276  0.945455  0.923077  0.912281  0.903226  0.888889  0.854839  0.836066  0.830508  0.825397  0.818182  0.807692  0.8  0.762712  0.761905  0.734375  0.733333  0.706897  0.654545  0.574074  0.561404  0.560606  0.476923  0.40678  0.272727  0.235294  0.107692  0.0677966 | 1  1  0.964286  0.851852  0.948276  0.690909  0.807692  0.807018  0.903226  0.666667  0.532258  0.819672  0.813559  0.730159  0.818182  0.346154  0.76  0.59322  0.507937  0.75  0.633333  0.637931  0.545455  0.518519  0.263158  0.545455  0.323077  0.474576  0.409091  0.215686  0.123077  0.101695 |

**Table S1: Fraction of orthologs in *Saccharomycetes* that have high levels of prion-like composition**

*** Column (1): Fraction of orthologs that have -log(fLPS P-value) ≥10.0**

**Column (2): Fraction of orthologs that have PLAAC PRD score ≥15.0**
