# Supplementary material for: Variable absorption of mutational trends by prion-forming domains during Saccharomycetes evolution
Source: PeerJ. 2020 Aug 6;8:e9669. doi: 10.7717/peerj.9669 (PMC7415223; doi:10.7717/peerj.9669)
Supplement: Supplemental Information 3 [file peerj-08-9669-s003.docx]

| 1. **Correlations of other prion formers across Saccharomycetes for fLPS P-value compositional bias**   **Table S2: Trends for other prion-formers †^(see footnote at end of document)^** | | | | | | | | | | | | | | | |
| --- | --- | --- | --- | --- | --- | --- | --- | --- | --- | --- | --- | --- | --- | --- | --- |
| **UniProtID +**  **UniProtName** | **N** | **% polyN+**  **polyQ** | | **% polyN** | | **% polyQ** | | **DNA GC%** | | **% in proteome with –log fLPS P-value ≥8.0** | | **% in proteome with –log fLPS P-value ≥10.0** | | **% in proteome with –log fLPS P-value ≥12.0** | |
| **Weighted (W) or Unweighted (UW)** |  | **W** | **UW** | **W** | **UW** | **W** | **UW** | **W** | **UW** | **W** | **UW** | **W** | **UW** | **W** | **UW** |
| P12383 PDR1_YEAST | 22 | 0.543* | 0.410 | 0.559* | 0.429* | 0.197 | 0.127 | -0.490* | -0.409 | 0.597* | 0.385 | 0.603* | 0.384 | 0.592* | 0.383 |
| P14907 NSP1_YEAST | 57 | 0.349* | 0.319 * | 0.382* | 0.350* | 0.074 | 0.068 | -0.384* | -0.321* | 0.374* | 0.336* | 0.350* | 0.319* | 0.326* | 0.304* |
| P18494 GLN3_YEAST*** | 58 | 0.836*** | 0.615 *** | 0.888  *** | 0.701  *** | 0.200 | 0.092 | -0.593*** | -0.397* | 0.867  *** | 0.666*** | 0.878*** | 0.698*** | 0.878*** | 0.723*** |
| P32770 NRP1_YEAST*** | 63 | 0.624*** | 0.553 *** | 0.681  *** | 0.592  *** | 0.115 | 0.172 | -0.482*** | -0.433  *** | 0.643  *** | 0.525*** | 0.657*** | 0.541*** | 0.676*** | 0.567*** |
| P32831 NGR1_YEAST | 55 | 0.178 | 0.212 | 0.081 | 0.138 | 0.259 | 0.247 | -0.236 | -0.264 | 0.153 | 0.197 | 0.158 | 0.203 | 0.155 | 0.201 |
| P38180 YBI1_YEAST*** | 27 | 0.647*** | 0.646 *** | 0.622  ** | 0.626  *** | 0.225 | 0.215 | -0.674*** | -0.687  *** | 0.575* | 0.536* | 0.554* | 0.516* | 0.537* | 0.503* |
| P38216 YBM6_YEAST | 4 | 0.000 | -0.237 | 0.000 | -0.316 | 0.000 | 0.210 | 0.000 | 0.292 | 0.000 | -0.214 | -0.000 | -0.206 | 0.000 | -0.169 |
| P38429 SAP30_YEAST* | 65 | 0.284* | 0.051 | 0.253* | 0.057 | 0.188 | 0.011 | -0.376* | -0.204 | 0.315* | -0.017 | 0.279* | -0.037 | 0.239 | -0.047 |
| P38691 KSP1_YEAST*** | 59 | 0.510*** | 0.460 *** | 0.715  *** | 0.639  *** | 0.092 | 0.082 | -0.480*** | -0.432  *** | 0.652  *** | 0.586*** | 0.660*** | 0.592*** | 0.663*** | 0.595*** |
| P40070 LSM4_YEAST*** | 59 | 0.386* | 0.335 * | 0.427  ** | 0.396* | 0.045 | 0.008 | -0.531*** | -0.472  *** | 0.509  *** | 0.421** | 0.480*** | 0.399* | 0.452*** | 0.379* |
| P40356 MED3_YEAST | 27 | 0.344 | 0.329 | 0.373 | 0.362 | 0.059 | 0.037 | -0.512* | -0.448* | 0.466* | 0.471* | 0.470* | 0.476* | 0.450* | 0.458* |
| P40956 GTS1_YEAST | 58 | 0.180 | 0.120 | 0.214 | 0.167 | -0.005 | -0.039 | -0.101 | -0.047 | 0.245 | 0.207 | 0.256 | 0.220 | 0.262* | 0.228 |
| P53894 CBK1_YEAST** | 59 | 0.163 | 0.187 | 0.028 | 0.049 | 0.340* | 0.361* | -0.126 | -0.126 | 0.123 | 0.093 | 0.107 | 0.087 | 0.088 | 0.079 |
| Q05166 NUP59_YEAST*** | 54 | 0.844*** | 0.821 *** | 0.870  *** | 0.837  *** | 0.200 | 0.266 | -0.563*** | -0.580  *** | 0.827  *** | 0.761*** | 0.843*** | 0.774*** | 0.858*** | 0.788*** |
| Q05672 RBS1_YEAST*** | 52 | 0.710*** | 0.626 *** | 0.761  *** | 0.704  *** | 0.126 | 0.098 | -0.585*** | -0.543  *** | 0.774  *** | 0.707*** | 0.787*** | 0.721*** | 0.793*** | 0.734*** |
| Q08925 MRN1_YEAST*** | 65 | 0.510*** | 0.393** | 0.448*** | 0.302* | 0.370* | 0.360* | -0.482*** | -0.439  *** | 0.515  *** | 0.376* | 0.478*** | 0.337* | 0.436*** | 0.300* |
| Q12139 YP022_YEAST*** | 51 | 0.422* | 0.433** | 0.357* | 0.370* | 0.317* | 0.319* | -0.477*** | -0.431  ** | 0.401* | 0.398* | 0.362* | 0.363* | 0.329* | 0.332* |
| Q12221 PUF2_YEAST** | 9 | 0.830* | 0.850* | 0.716* | 0.780* | 0.887** | 0.874* | -0.409 | -0.568 | 0.571 | 0.698* | 0.624 | 0.723* | 0.711* | 0.769* |
| Q12224 RLM1_YEAST*** | 30 | 0.629*** | 0.556** | 0.622*** | 0.564** | 0.378* | 0.324 | -0.371* | -0.285 | 0.699*** | 0.600*** | 0.729*** | 0.627*** | 0.738*** | 0.638*** |
| Q12361 GPR1_YEAST | 59 | 0.280* | 0.285* | 0.271* | 0.273* | 0.143 | 0.155 | -0.323* | -0.312* | 0.305* | 0.307* | 0.289* | 0.291* | 0.270* | 0.271* |
| Q99383 HRP1_YEAST | 57 | 0.067 | -0.046 | 0.094 | -0.064 | -0.017 | 0.023 | -0.132 | -0.073 | 0.130 | -0.002 | 0.134 | -0.009 | 0.123 | -0.026 |

| 1. **Correlations of other prion formers across Saccharomycetes for PLAAC PRDscore for prion-like composition** | | | | | | | | | | | | | | | |
| --- | --- | --- | --- | --- | --- | --- | --- | --- | --- | --- | --- | --- | --- | --- | --- |
| **UniProtID +**  **UniProtName** | **N** | **% polyN+**  **polyQ** | | **% polyN** | | **% polyQ** | | **DNA GC%** | | **% of proteome with PLAAC PRDscore >0.0** | | **% of proteome with PLAAC PRDscore ≥15.0** | | **% of proteome with PLAAC PRDscore ≥30.0** | |
| **Weighted (W) or Unweighted (UW)** |  | **W** | **UW** | **W** | **UW** | **W** | **UW** | **W** | **UW** | W | **UW** | **W** | **UW** | **W** | **UW** |
| P12383 PDR1_YEAST | 22 | 0.356 | -0.015 | 0.283 | -0.027 | 0.353 | 0.027 | -0.111 | 0.052 | 0.332 | -0.197 | 0.371 | -0.154 | 0.387 | -0.231 |
| P14907 NSP1_YEAST | 57 | 0.177 | 0.150 | 0.149 | 0.124 | 0.134 | 0.116 | -0.280* | -0.218 | 0.225 | 0.228 | 0.168 | 0.168 | 0.084 | 0.086 |
| P18494 GLN3_YEAST*** | 58 | 0.764  *** | 0.573  *** | 0.866*** | 0.705*** | 0.059 | -0.021 | -0.428** | -0.282* | 0.681*** | 0.573*** | 0.745*** | 0.631*** | 0.810*** | 0.699*** |
| P32770 NRP1_YEAST*** | 63 | 0.478  *** | 0.354* | 0.546*** | 0.391* | 0.034 | 0.085 | -0.348* | -0.250* | 0.478*** | 0.314* | 0.522*** | 0.364* | 0.578*** | 0.390* |
| P32831 NGR1_YEAST | 55 | 0.098 | 0.133 | -0.060 | -0.004 | 0.296* | 0.285* | -0.045 | -0.099 | 0.089 | 0.141 | 0.111 | 0.154 | 0.137 | 0.151 |
| P38180 YBI1_YEAST | 27 | 0.083 | 0.071 | 0.083 | 0.058 | 0.015 | 0.056 | -0.198 | -0.206 | 0.006 | -0.149 | 0.068 | -0.074 | 0.146 | -0.091 |
| P38216 YBM6_YEAST | 4 | 0.000 | -0.237 | 0.000 | -0.316 | -0.000 | 0.210 | 0.000 | 0.292 | 0.000 | -0.208 | 0.000 | -0.121 | 0.000 | -0.141 |
| P38429 SAP30_YEAST | 65 | 0.139 | -0.030 | 0.102 | -0.026 | 0.141 | -0.021 | -0.084 | -0.062 | 0.169 | -0.150 | 0.160 | -0.133 | 0.120 | -0.182 |
| P38691 KSP1_YEAST** | 59 | 0.264* | 0.229 | 0.415** | 0.339* | -0.009 | 0.013 | -0.284* | -0.223 | 0.264* | 0.195 | 0.210 | 0.162 | 0.196 | 0.101 |
| P40070 LSM4_YEAST*** | 59 | 0.514  *** | 0.437** | 0.518*** | 0.461*** | 0.185 | 0.133 | -0.570*** | -0.502*** | 0.607*** | 0.448*** | 0.540*** | 0.406** | 0.468*** | 0.336* |
| P40356 MED3_YEAST | 27 | 0.315 | 0.314 | 0.360 | 0.366 | -0.013 | -0.037 | -0.538* | -0.492* | 0.472* | 0.484* | 0.410* | 0.431* | 0.407* | 0.440* |
| P40956 GTS1_YEAST | 58 | 0.147 | 0.121 | 0.175 | 0.147 | -0.005 | 0.004 | -0.083 | -0.075 | 0.309* | 0.273* | 0.260* | 0.237 | 0.248 | 0.231 |
| P53894 CBK1_YEAST | 59 | 0.083 | 0.085 | 0.020 | 0.031 | 0.161 | 0.148 | -0.156 | -0.145 | 0.199 | 0.148 | 0.131 | 0.105 | 0.088 | 0.056 |
| Q05166 NUP59_YEAST*** | 54 | 0.545  *** | 0.523  *** | 0.539*** | 0.512*** | 0.189 | 0.220 | -0.308* | -0.365* | 0.550*** | 0.455** | 0.544*** | 0.454** | 0.541*** | 0.420** |
| Q05672 RBS1_YEAST*** | 52 | 0.505  *** | 0.434** | 0.530*** | 0.488*** | 0.118 | 0.065 | -0.408** | -0.382* | 0.631*** | 0.566*** | 0.603*** | 0.538*** | 0.626*** | 0.561*** |
| Q08925 MRN1_YEAST*** | 65 | 0.443  *** | 0.333* | 0.371* | 0.241 | 0.358* | 0.338* | -0.376* | -0.354* | 0.314* | 0.269* | 0.313* | 0.257* | 0.236 | 0.168 |
| Q12139 YP022_YEAST | 51 | 0.146 | 0.182 | 0.097 | 0.136 | 0.168 | 0.171 | -0.110 | -0.123 | 0.196 | 0.229 | 0.159 | 0.196 | 0.097 | 0.120 |
| Q12221 PUF2_YEAST** | 9 | -0.335 | -0.564 | -0.531 | -0.666 | 0.188 | -0.216 | 0.903** | 0.862* | 0.120 | -0.402 | 0.024 | -0.411 | 0.183 | -0.387 |
| Q12224 RLM1_YEAST*** | 30 | 0.407* | 0.363* | 0.119 | 0.109 | 0.627*** | 0.546* | 0.060 | 0.072 | 0.486* | 0.427* | 0.541* | 0.475* | 0.515* | 0.424* |
| Q12361 GPR1_YEAST | 59 | -0.025 | -0.017 | -0.022 | -0.013 | -0.017 | -0.017 | -0.011 | -0.015 | -0.007 | 0.002 | -0.004 | -0.000 | -0.075 | -0.070 |
| Q99383 HRP1_YEAST | 57 | 0.106 | -0.082 | 0.106 | -0.109 | 0.056 | 0.031 | -0.141 | -0.042 | 0.249 | 0.099 | 0.204 | 0.029 | 0.161 | -0.040 |

**†: The asterisk notations for correlations are as in Tables 1 and 2, with asterisks also used in column 1 attached to the UniProt names, to indicate the strongest level of correlation observed for each protein.**

**N = number of sequences.**

**'W' stands for weighted correlation and 'UW' for unweighted correlation.**

**'% polyN' is the percentage of poly-asparagine out of all the residues in a proteome.**

**'% polyQ' is the percentage of poly-glutamine out of all the residues in a proteome.**

**'DNA GC%' is the percentage of guanidine+cytidine in the DNA.**

**'% of proteome -log fLPS P-value ≥X' is the percentage of proteins in a proteome that have compositional bias for N and/or Q residues determined by -log of the P-value determined by the fLPS program being ≥X.**

**'% of proteome PLAAC PRDscore ≥X or >X' is the percentage of proteins in a proteome that have prion-like composition determined by a PRDscore from the PLAAC program being ≥X or >X.**
