# Supplementary material for: Variable absorption of mutational trends by prion-forming domains during Saccharomycetes evolution
Source: PeerJ. 2020 Aug 6;8:e9669. doi: 10.7717/peerj.9669 (PMC7415223; doi:10.7717/peerj.9669)
Supplement: Supplemental Information 4 [file peerj-08-9669-s004.docx]

|  | | | | | | | | | |
| --- | --- | --- | --- | --- | --- | --- | --- | --- | --- |
| **Table S3: Analysis of PLAAC data using Spearman rank correlation coefficients †** | | | | | | | | | |
| **Protein**  **(Number of orthologs in brackets)** | **%N in proteome** | **%Q in proteome** | **%poly-N** | **%poly-Q** | **%poly-Q**  **+**  **%poly-N** | **DNA GC%** | **Fraction of prion-like proteins in the proteome by PLAAC PRDscore** | | |
|  |  |  |  |  |  |  | **>0.0** | **≥15.0** | **≥30.0** |
| **Known amyloid-based prions in *S. cerevisiae*** | | | | | | | | | |
| **Sup35**  **P05453 (62)** | 0.471*** | 0.241 | 0.538*** | 0.503*** | 0.555*** | –0.414** | 0.581*** | 0.579*** | 0.534*** |
| **Swi1 ††**  **P09547 (56)** | 0.397** | –0.254 | 0.376** | 0.018 | 0.300* | –0.445** | 0.403** | 0.391* | 0.354* |
| **Cyc8**  **P14922 (61)** | 0.309* | 0.299* | 0.415** | 0.411** | 0.438*** | –0.299* | 0.496*** | 0.467*** | 0.388* |
| **Ure2**  **P23202 (66)** | 0.551*** | 0.048 | 0.589*** | 0.289* | 0.534*** | –0.478*** | 0.380** | 0.377* | 0.254* |
| **Rnq1 †**  **P25367 (26)** | 0.368 | –0.452* | 0.331 | –0.166 | 0.118 | –0.189 | 0.153 | 0.098 | –0.033 |
| **Mot3 †**  **P54785 (25)** | 0.181 | –0.365 | 0.287 | 0.352 | 0.228 | –0.140 | 0.018 | 0.130 | –0.044 |
| **Nu100 †**  **Q02629 (11)** | 0.292 | 0.137 | 0.255 | 0.118 | 0.182 | –0.041 | 0.351 | 0.187 | –0.023 |
| **Pin3 †**  **Q06449 (55)** | –0.008 | 0.193 | 0.040 | 0.182 | 0.089 | –0.006 | –0.040 | –0.010 | 0.013 |
| **Other prion-forming domains discussed in the text** | | | | | | | | | |
| **New1 ††**  **Q08972 (63)** | 0.387* | 0.223 | 0.377* | 0.218 | 0.347* | –0.416** | 0.427** | 0.410** | 0.345* |
| **Pub1**  **P32588 (62)** | 0.339* | 0.357* | 0.467*** | 0.559*** | 0.530*** | –0.349* | 0.483*** | 0.501*** | 0.469*** |
| † The labelling and colour-coding are as in Table 2. | | | | | | | | | |
