# Supplementary material for: Variable absorption of mutational trends by prion-forming domains during Saccharomycetes evolution
Source: PeerJ. 2020 Aug 6;8:e9669. doi: 10.7717/peerj.9669 (PMC7415223; doi:10.7717/peerj.9669)
Supplement: Supplemental Information 5 [file peerj-08-9669-s005.docx]

**Table S4: Analysis using PLAAC LLR score instead of PRD score**

| **Correlations of percentage of (poly-N + poly-Q) versus PLAAC LLR score** | | | |
| --- | --- | --- | --- |
| **UniProtID and UniProtName** | **Pearson R**  **correlation** | **# of orthologs** | **P-values and significance †** |
| P05453 ERF3_YEAST | 0.159 | 62 | NS (*) |
| P09547 SWI1_YEAST | 0.328 | 56 | 0.0136* (**) |
| P14922 CYC8_YEAST | 0.394 | 61 | 0.0017** (***) |
| P23202 URE2_YEAST | 0.493 | 66 | 0.00003*** (***) |
| P25367 RNQ1_YEAST | -0.048 | 26 | NS (NS) |
| P32432 SFP1_YEAST | -0.042 | 64 | NS (NS) |
| P32588 PUB1_YEAST | 0.569 | 62 | <0.00001*** (***) |
| P54785 MOT3_YEAST | -0.043 | 25 | NS (NS) |
| Q02629 NU100_YEAST | 0.100 | 11 | NS (NS) |
| Q06449 PIN3_YEAST | 0.080 | 55 | NS (NS) |
| Q08972 NEW1_YEAST | 0.282 | 63 | 0.0254* (*) |

| **DNA GC% versus PLAAC LLR score** | | | |
| --- | --- | --- | --- |
| **UniProtID and UniProtName** | **Pearson R**  **correlation** | **# of orthologs** | **P-values and significance †** |
| P05453 ERF3_YEAST | -0.234 | 62 | NS (*) |
| P09547 SWI1_YEAST | -0.380 | 56 | 0.0039** (***) |
| P14922 CYC8_YEAST | -0.410 | 61 | 0.001** (***) |
| P23202 URE2_YEAST | -0.479 | 66 | 0.00005*** (***) |
| P25367 RNQ1_YEAST | 0.114 | 26 | NS (NS) |
| P32432 SFP1_YEAST | -0.020 | 64 | NS (NS) |
| P32588 PUB1_YEAST | -0.390 | 62 | 0.0017** (***) |
| P54785 MOT3_YEAST | 0.101 | 25 | NS (NS) |
| Q02629 NU100_YEAST | -0.312 | 11 | NS (NS) |
| Q06449 PIN3_YEAST | -0.041 | 55 | NS (NS) |
| Q08972 NEW1_YEAST | -0.382 | 63 | 0.002** (**) |

| **Fraction of proteome with PLAAC LLR score ≥15.0 *versu*s PLAAC LLR score** | | | |
| --- | --- | --- | --- |
| **UniProtID and UniProtName** | **Pearson R**  **correlation** | **# of orthologs** | **P-values and significance †** |
| P05453 ERF3_YEAST | 0.193 | 62 | NS (**) |
| P09547 SWI1_YEAST | 0.379 | 56 | 0.004** (***) |
| P14922 CYC8_YEAST | 0.490 | 61 | 0.00006*** (***) |
| P23202 URE2_YEAST | 0.484 | 66 | 0.00004*** (***) |
| P25367 RNQ1_YEAST | -0.063 | 26 | NS (NS) |
| P32432 SFP1_YEAST | -0.001 | 64 | NS (NS) |
| P32588 PUB1_YEAST | 0.580 | 62 | <0.00001*** (***) |
| P54785 MOT3_YEAST | -0.062 | 25 | NS (NS) |
| Q02629 NU100_YEAST | 0.376 | 11 | NS (NS) |
| Q06449 PIN3_YEAST | 0.040 | 55 | NS (NS) |
| Q08972 NEW1_YEAST | 0.329 | 63 | 0.009* (**) |

**†: The P-value is labelled with asterisks as follows:**

*** for 0.05≥P<0.005**

**** for 0.005≥P<0.0005**

***** for P<0.0005**

**with NS for non-significant. In brackets, are the corresponding**

**labels when using the PLAAC PRDscore.**
